# How contact patterns during the COVID-19 pandemic are related to pre-pandemic contact patterns and mobility trends

**DOI:** 10.1186/s12879-023-08369-8

**Published:** 2023-06-16

**Authors:** Adrien Lajot, James Wambua, Pietro Coletti, Nicolas Franco, Ruben Brondeel, Christel Faes, Niel Hens

**Affiliations:** 1 Department of Epidemiology and Public Health, Sciensano, Brussels, Belgium; 2 Data Science Institute, I-BioStat, University of Hasselt, Hasselt, Belgium; 3 Namur Institute for Complex Systems (naXys) and Department of Mathematics, University of Namur, Namur, Belgium; 4 Centre for Health Economic Research and Modelling Infectious Diseases, Vaccine and infectious disease institute, University of Antwerp, Antwerp, Belgium

**Keywords:** COVID-19, SARS-CoV-2, Contact patterns, Mobility trends, Time-varying effect model

## Abstract

**Background:**

Non-pharmaceutical interventions (NPIs) were adopted in Belgium in order to decrease social interactions between people and as such decrease viral transmission of SARS-CoV-2. With the aim to better evaluate the impact of NPIs on the evolution of the pandemic, an estimation of social contact patterns during the pandemic is needed when social contact patterns are not available yet in real time.

**Methods:**

In this paper we use a model-based approach allowing for time varying effects to evaluate whether mobility and pre-pandemic social contact patterns can be used to predict the social contact patterns observed during the COVID-19 pandemic between November 11, 2020 and July 4, 2022.

**Results:**

We found that location-specific pre-pandemic social contact patterns are good indicators for estimating social contact patterns during the pandemic. However, the relationship between both changes with time. Considering a proxy for mobility, namely the change in the number of visitors to transit stations, in interaction with pre-pandemic contacts does not explain the time-varying nature of this relationship well.

**Conclusion:**

In a situation where data from social contact surveys conducted during the pandemic are not yet available, the use of a linear combination of pre-pandemic social contact patterns could prove valuable. However, translating the NPIs at a given time into appropriate coefficients remains the main challenge of such an approach. In this respect, the assumption that the time variation of the coefficients can somehow be related to aggregated mobility data seems unacceptable during our study period for estimating the number of contacts at a given time.

**Supplementary Information:**

The online version contains supplementary material available at 10.1186/s12879-023-08369-8.

## Background

Since the beginning of the COVID-19 pandemic, Belgium has adopted several Non-Pharmaceutical Interventions (NPIs), including school closures, mandatory homeworking and restrictions on the number of contacts, in order to curb the increase in hospital admissions and preserve hospital capacity. As NPIs have huge social [1, 2] and economic [2, 3] costs, a good comprehension of transmission dynamics under these conditions is necessary to predict which NPIs could be the most effective in slowing down the transmission of the virus.

A method commonly used today to study the transmission dynamics of airborne pathogens is the use of the so-called social contact hypothesis [4]. Under this hypothesis, it is assumed that transmission rates are proportional to contact rates. The social contact hypothesis, adapted to account for age specific differences, has been used in several models to predict and understand the COVID-19 pandemic in Belgium [5–8]. These authors have used pre-pandemic contact patterns [9–11], i.e. data collected before the pandemic when NPIs were not in place, to investigate the possible dynamics of the COVID-19 pandemic given no social contact patterns were available in the initial phase of the pandemic.

Although several studies have demonstrated the ability of NPIs to reduce contacts [12–17], there is a need to evaluate social contact patterns during the pandemic when social contact patterns are not yet available in real time. In this regard, it is necessary to assess whether social contact patterns under specific NPIs can be inferred from available data, such as location-specific pre-pandemic contact patterns and freely available aggregated mobility data. This is not trivial as not only the NPIs but also the epidemiological context of the pandemic and the weariness against the NPIs are likely to influence people’s social behaviour. A study conducted in Belgium between April 2020 and April 2021 shows that risk perception and perceived effectiveness of NPIs play a role in social contact patterns [18]. Another study covering 16 European countries between December 2020 and September 2021 shows similar results [19]. In the absence of readily available data, some models have assumed that social contact patterns during and after the lockdown can be approximated by a linear combination of pre-pandemic social contact patterns in different locations (e.g. contacts at home, at work, etc) [5–8]. Others have assumed that social contact patterns are related to the mobility of individuals, and therefore e.g. Google mobility data could be used to scale pre-pandemic contact matrices to pandemic contact matrices [20, 21]. Tomori et al. [22] found that both contact behaviour and mobility behaviour are needed to capture the full aspect of the transmission dynamics of COVID-19 in Germany between April 2020 and June 2020. Instead of estimating contact patterns, other studies have inferred complex contact networks from the GPS data of mobile phones of a fraction of the population [23–25]. Although these high-resolution data appear to be more effective in modeling transmission dynamics [23], they may lead to privacy issues and GDPR restrictions. For this reason, they are likely to be less readily available than publicly available aggregated mobility data. In this paper, we investigate whether social contact patterns under specific NPIs can be obtained from readily available data, i.e., both pre-pandemic contact patterns and aggregated mobility data.

This study is based on two social contact surveys, namely the CoMix survey conducted in Belgium during the pandemic [12, 26] and a survey conducted in Flanders (Belgium) before the pandemic in 2010-2011 [10, 11]. In addition Google mobility data is used [27]. The Belgian CoMix survey has monitored social contact patterns, stratified by age groups, during more than 40 waves of data collection during the COVID-19 pandemic [12] at different conceptual locations (e.g. home, work, school, leisure, transport and other places). For the present work, CoMix data collected between November 11, 2020 and July 4, 2022 were used. The 2010-2011 study [10, 11] surveyed social contacts in Flanders (Belgium) before the pandemic at the same conceptual locations as CoMix. Mobility data from Google is openly available, and quantifies the change in the number of visitors to different types of locations over time relative to a baseline (e.g. retail and recreation, grocery and pharmacy, parks, transit stations, workplaces). The change in time spent at home is also quantified. For the current article, we used the change in the number of visitors to transit stations collected between November 11, 2020 and July 4, 2022 [27].

A time varying effect model [28] was built to describe the relationship between the social contact patterns observed during the pandemic and pre-pandemic social contact patterns. To examine whether mobility can explain the temporal variation in this relationship, a second time-varying model was constructed that considered the interaction between mobility and pre-pandemic data.

This paper is structured as follows. The data used and the statistical methodology are covered in Methods section. The results are given in Result section. Discussion section discusses the results and its implications.

## Methods

### Data

**Fig. 1:**
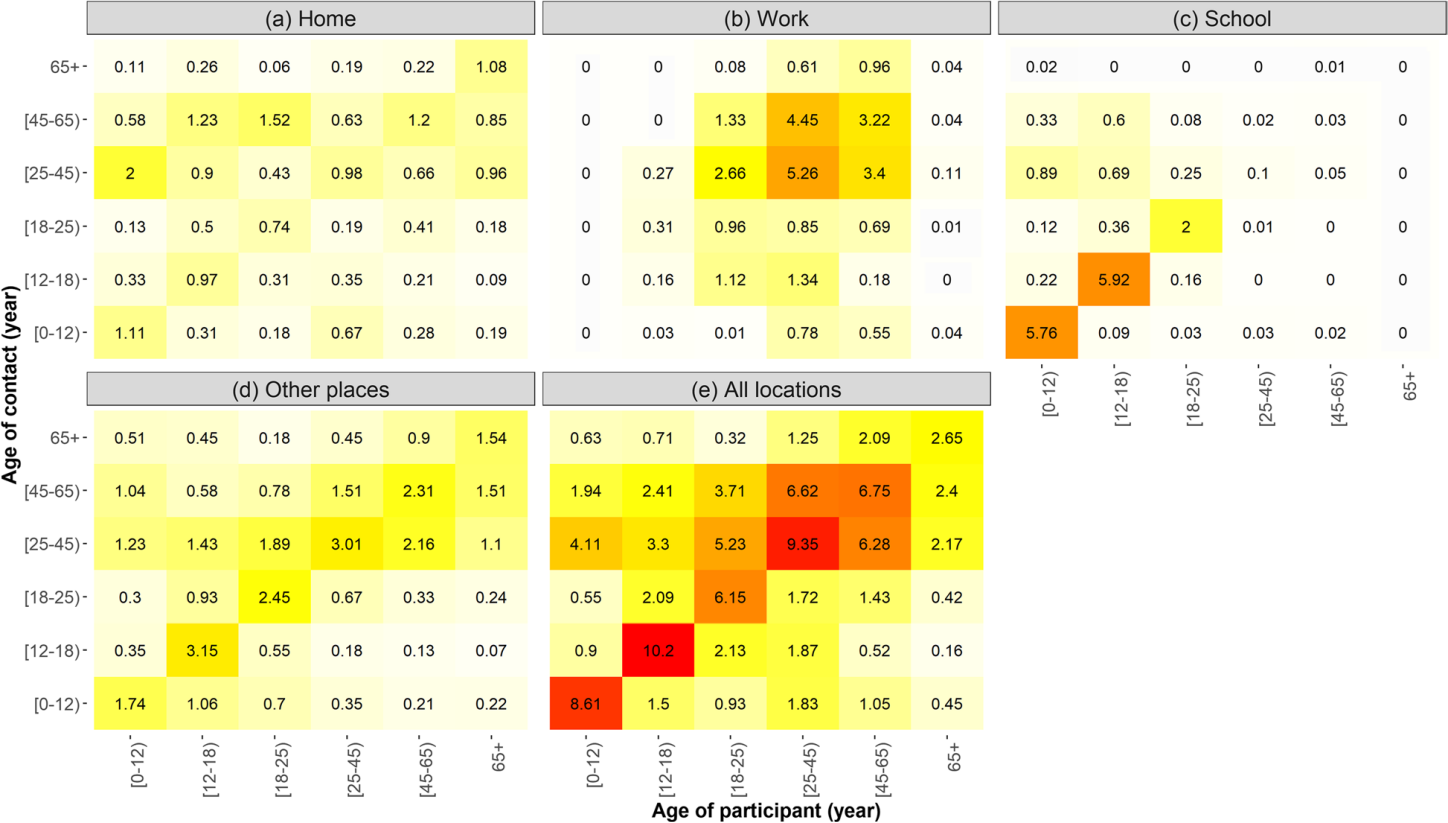
Contact matrices (average number of contacts per day) during pre-pandemic times at different locations (at home (**a**), work (**b**), school (**c**), other places (**d**) and at all locations (**e**)) based on a survey conducted in 2010-2011  [10, 11] and generated by the Socrates tool [7, 29]

*Social contact patterns before the pandemic*: The pre-pandemic contact patterns were obtained from a cross-sectional diary-based survey of social contacts conducted in 2010-2011 in Brussels and Flanders, Belgium. This survey can be assumed representative of the whole Belgian population. Indeed a similar survey conducted in 2006 considering all the Belgian population showed no statistical differences in social contacts rates with this survey which considered only Flanders and Brussels (68% of the Belgian population on January 1, 2023) [10]. In the 2010 survey, 1759 people were asked to detail their social contacts for a single day [10, 11]. Participants were asked to describe all contacts made on a given day, the type of contact, the location of the contact, and the age of the person contacted, with a contact defined as a face-to-face conversation of at least a few words, or a skin-to-skin contact. This survey has been used in some recent epidemiological models [5–8] and is described in [10] and [11]. Based on this survey, the age-specific contact patterns at home, work, transport, leisure, school and other places amongst individuals in age groups [0-12), [12,18), [18,25), [25,45) [45,65) and 65+ were generated using the Socrates tool. This tool allows the extraction and generation of social contact matrices [7, 29]. Pre-pandemic contacts at leisure and transport were categorised as contacts at other places. This is summarised in 4 contact matrices and visualised in Fig. 1. A fifth contact matrix depicting the sum of the 4 location-specific matrices is also shown in Fig. 1.

**Fig. 2:**
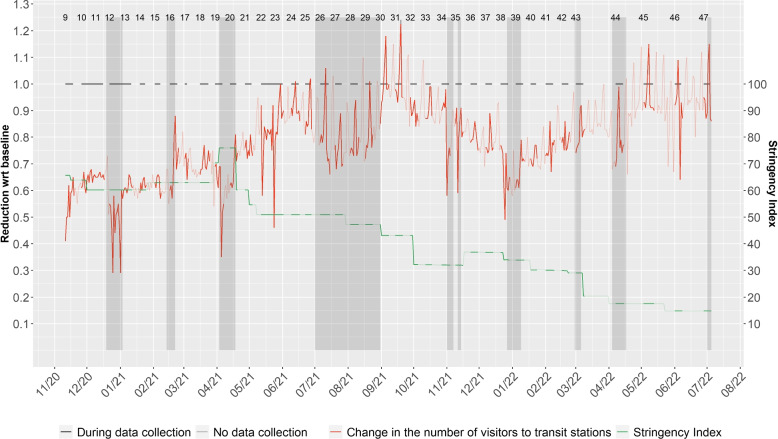
Stringency index from Oxford University (green) [30] and the relative change in the number of visitors to transit stations (red) compared to the baseline period (January 3 - February 6), based on Google mobility data [27]. Full color refers to period with CoMix data collection while lighter color refers to out of CoMix data collection. Holiday periods are depicted in the shaded areas. Black lines depict the periods corresponding to the different CoMix waves [12, 26]

*Social contact patterns during the pandemic*: The pandemic social contact patterns data were obtained from the CoMix survey, a longitudinal online survey on social contact patterns set up during the COVID-19 pandemic in different European countries [12, 26]. For the present study, we only utilize datasets collected in Belgium. These datasets are representative of the Belgian population in term of age, gender and region of residence. The definition for contact were similar as the survey of 2010, i.e. an in-person conversation of at least a few words, or a skin-to-skin contact. Based on this survey, the age-specific contact patterns amongst individuals in age groups [0-12), [12,18), [18,25), [25,45) [45,65) and 65+ were obtained at 39 different waves of the survey conducted between the period spanning between November 11, 2020 and July 04, 2022 [12, 26]. The first eight waves of data collection in the CoMix survey did not include children and are therefore not considered in this work. The 39 different waves of CoMix data collection considered in this work therefore correspond to waves 9 to 47. This is summarised with 39 contact matrices and visualised in (Additional file 1: Fig. S5). The time period corresponding to each CoMix data collection wave can be visualised in Fig. 2. Age specific contact patterns at the same locations as in the pre-pandemic survey were also obtained in order to calculate the number of contacts at each location. The selected waves of data collection cover a period during which NPIs were in place. However, the stringency index from the University of Oxford [30] shows that the strictness of ‘lockdown style’ varied over time (Fig. 2). The average interval between two consecutive waves of data collection was 2 weeks between November 11, 2020 and March 08, 2022 and 4 weeks between March 08, 2022 and July 04, 2022. These intervals were not dependent on the NPI in place. Each individual was invited every 2 weeks (November 2020 - March 2022) and 4 weeks (March 2022-July 2022).

*Google mobility data*: Google provides data describing the change in the number of visitors to a specific location relative to the amount of total visitors to the same location with respect to the baseline, which is the median value from the 5-week period Jan 3 - Feb 6, 2020 [27]. For example, a value of 0.80 for a specific place would then correspond to a decrease of 20% in the number of visitors to that place compared to the baseline period. The baseline period corresponds to a relative change of 1. The available settings are retail and recreation, grocery and pharmacy, parks, transit stations, workplaces, and residential. The residential category refers to a change in time spent at home rather than a change in the number of visitors. We consider that changes in the number of visitors to transit stations imply changes in various locations. For this reason we consider that this setting is the most suitable to predict the overall change in contact patterns. Corresponding to the times of the CoMix waves, the mean change in the number of visitors to transit stations were computed. Figure 2 shows the trend between November 11, 2020 and July 4, 2022. It can be seen that between November, 2020 and April, 2020, the number of visitors to transit stations remained at a relatively low level as compared to the pre-pandemic level (Fig. 2). From April, 2021 onwards, an increase in the number of visitors to transit stations followed by a decrease during the summer holiday and a stabilisation to a level close to the pre-pandemic level until November 2021 was observed. From November 2021, the total number of visitors decreases until February 2022 and increases again until July 2022. It is worth mentioning that holiday periods are always associated with a decrease in the number of visitors to transit stations. However, the absolute level of the change differs between the different holiday periods.

### Model

The main interest is in the relationship between contact patterns during the pandemic and before the pandemic. As this relationship is expected to be time-dependent, we employed a time varying effect model [28] considering a linear combination of pre-pandemic contacts at different locations [10, 11]. The dependent variable $Y_{ijt}$ is the average number of contacts for participants of age *j* with individuals of age *i* at time *t*, where $t=9,\ldots , 47$ refers to the 39 waves of the CoMix survey considered in this work [12, 26]. The average number of pre-pandemic social contacts at home, work, school and other places were used as explanatory variables. Let $Z^k_{ij}$ denotes the pre-pandemic average number of contacts for participants of age *j* with individuals of age *i* at location *k*, with *k* corresponding to home, work, school and other places. We then model $Y_{ijt}$ as:

1 $$\begin{aligned} Y_{ijt}\sim & {} N(\mu _{ijt},\sigma ^2)\end{aligned}$$

2 $$\begin{aligned} \mu _{ijt}= & {} \beta _{1}(t) Z_{ij}^{home} + \beta _{2}(t) Z_{ij}^{work} + \beta _{3}(t) Z_{ij}^{school} + \beta _{4}(t) Z_{ij}^{other} \end{aligned}$$

where $\beta _{p}(t)$ ($p=1,2,3,4$), refers to the 4 coefficient functions. It consists of smooth functions in time, modelled as a cubic P-spline with 10 knots. The chosen parameters (i.e. degree and number of knots) of the coefficient functions were the default parameters as recommended by [31]. Given that the CoMix survey is a longitudinal survey, observations are not independent from wave to wave. In order to take this correlation into account, robust standard errors were estimated.

The coefficient functions for each location can be interpreted as the evolution over time of the expected change in the total number of contacts between two age groups during the pandemic resulting from a change of one contact between those age groups at that locations in the pre-pandemic period. For example, a value of 0.5 at a given location would signify that one additional contact between two age groups at that location in the pre-pandemic period will result in a total of 0.5 additional contacts between those two age groups during the pandemic. As pre-pandemic value are expected to be larger than pandemic value, coefficient functions are expected to be estimated below 1. However, coefficients value close to 1 or higher than 1 are also possible. The particular case where the coefficient function at a given location is close to 1 could mean that a given change in that location at pre-pandemic time will result in a similar change in the expected total number of contacts during the pandemic. This could correspond to locations where social contact patterns are similar to the pre-pandemic period, such as home or school outside the holiday period. This could also be due to a compensatory behavior i.e. spending less time at work or school could imply meeting up with more people at home.

Some models have assumed that mobility can be used to scale pre-pandemic contact matrices to pandemic contact matrices. To test this assumption, a second time varying effect model has been explored considering a linear combination of pre-pandemic contacts at different locations in interaction with a proxy for mobility, namely the change in the number of visitors to transit stations with respect to the baseline period. Interest is in the ability or inability of mobility to reduce the variability of the smoothing functions $\beta _{p} (t)$. It is worth mentioning that in this model, we are interested in the variability of $\beta _{p} (t)$ over time rather than $\beta _{p} (t)$ itself. Let $T_t$ denotes mobility defined as the relative change in number of visitors to transit stations at time *t* as compared to the pre-pandemic period, based on Google mobility data [27]. The other parameters in this model are the same as in the first model. This second model can be summarised as follows:

3 $$\begin{aligned} Y_{ijt}\sim & {} N(\mu _{ijt},\sigma ^2)\end{aligned}$$

4 $$\begin{aligned} \mu _{ijt}= & {} \beta _{1}(t) T_{t} Z_{ij}^{home} + \beta _{2}(t) T_{t} Z_{ij}^{work} + \beta _{3}(t) T_{t} Z_{ij}^{school} + \beta _{4}(t) T_{t} Z_{ij}^{other} \end{aligned}$$

The time varying effect models [28] were fitted using the SAS macro tvem [31] version 3.1.1 in SAS version 9.4.

## Result

**Fig. 3:**
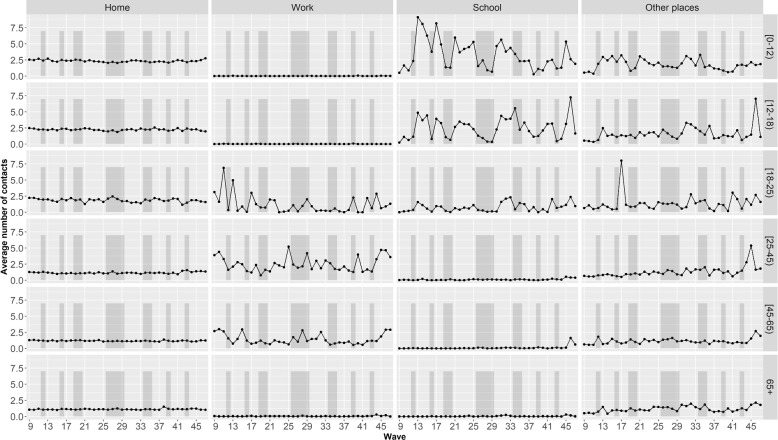
Evolution over time of the average number of contacts in a day during the pandemic at different locations (at home, work, school and other places) by age groups based on the CoMix survey [12, 26]. Holiday periods are depicted in the shaded areas

The evolution over time of the average number of contacts for each age groups at different locations is depicted in Fig. 3. Children (i.e people aged 0 to 17 years old) made more contacts than adults (i.e. people aged 18 to 64 years old) and elderly people (i.e. people aged 65+ years old) at all locations. For obvious reasons, children had no contacts at work, and adults had hardly any contacts at school. At home, the average number of contacts remained stable for all age categories throughout the study period, with younger age categories making more contacts than older age categories. Outside the holiday periods, children made more contacts at school compared to home and other places. The average number of contacts for children (i.e. people aged 0 to 17 years old) and students (i.e. people aged 18-25 years old) at school is very sensitive to the holiday period. However, when comparing the non-holidays periods, the average number of contacts is not equal between them. Moreover, students made fewer contacts at school than children. At work, the number of contacts made by adults (i.e. people aged 18 to 64 years old) varied over time, without any obvious trends. Finally, the graphs for other places suggest that there is a small positive trend in the number of contacts, except for the youngest children (i.e. people aged 0 to 11 years old). Moreover, within the school going age categories (i.e. people aged 0 to 24 years old), it can be noticed that there is more variability in the trend.

**Fig. 4:**
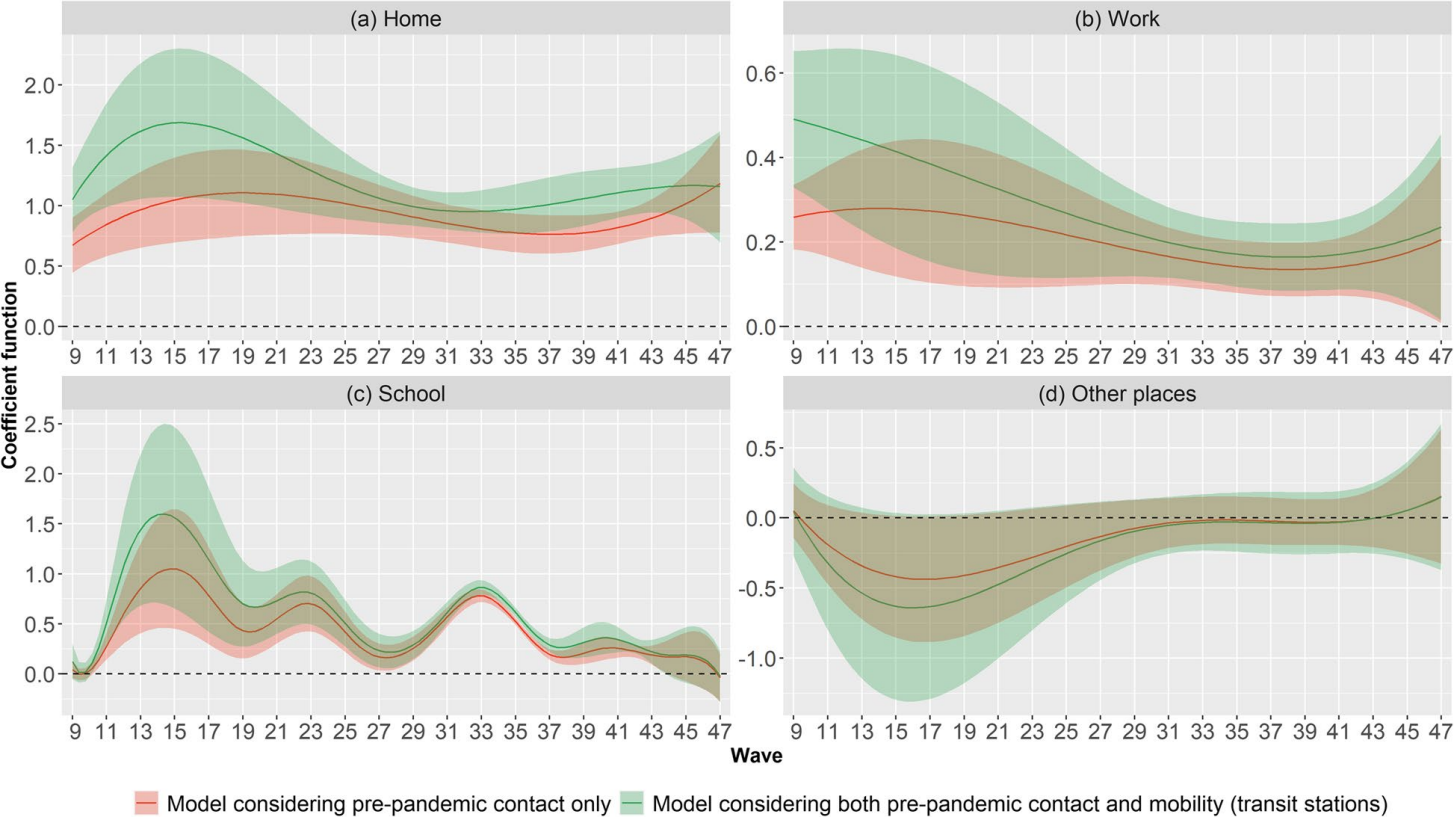
Coefficient functions of the time-varying coefficient models considering only pre-pandemic contacts (red) and considering both pre-pandemic contacts and Google mobility data at transit stations (green)

Figure 4 depicts the coefficient functions of the time varying coefficient models considering only pre-pandemic contacts (red) and considering mobility in addition (green). Concerning the model considering only pre-pandemic contacts, the coefficient functions can be interpreted as the evolution over time of the expected change in the total number of contacts during the pandemic resulting from a change of one contact at a given location at pre-pandemic time. As pre-pandemic contacts are likely to be larger than contacts during the pandemic, a value below 1 of the coefficient functions could be expected. However, a location with a coefficient function close to 1 or higher than 1 could be interpreted as a location in which social contact patterns are similar to the pre-pandemic period, or in locations subject to compensatory behavior (see Methods section). Figure 4a shows that at home it increased slightly for the first waves (i.e. from wave 9 to wave 20). It then followed a slight decreasing trends until wave 36 and then increased again. Throughout the study period, coefficient functions are close to 1. However, it is never statistically higher than 1. Figure 4b shows that a slight decreasing trend can be observed in the expected change in the total number of contacts during the pandemic resulting from a change of one contact at work. However, it remained lower than 1 and at a lower level than the one considering pre-pandemic contacts at home (Fig. 4a) and school outside holiday periods (Fig. 4c). At school the temporal trend of the coefficient function was more marked than at any other location. It followed a cyclic pattern between waves 9 and 36 and then stabilised from wave 37 onward. In November 2020 (i.e. waves 9 and 10), during the Easter 2021 (i.e. waves 19 and 20) and the summer holidays 2021 (i.e. from wave 26 to wave 30) as well as during the year 2022 (i.e. from wave 37 to wave 47), a shift of one contact at school at pre-pandemic time resulted in a smaller expected change in the total number of contacts during the pandemic than outside those periods. Outside holiday periods, the expected change was close to 1 and fairly similar to that for pre-pandemic contacts at home (Fig. 4a). Pre-pandemic contacts at other places (Fig. 4c) was not statistically associated with the total number of contacts during the pandemic throughout the study period.

Considering the time varying coefficient model using mobility data in addition to pre-pandemic contact resulted in larger variations in the time-varying coefficients for all locations. Moreover, it also added more uncertainty in the time-varying coefficients.

**Fig. 5:**
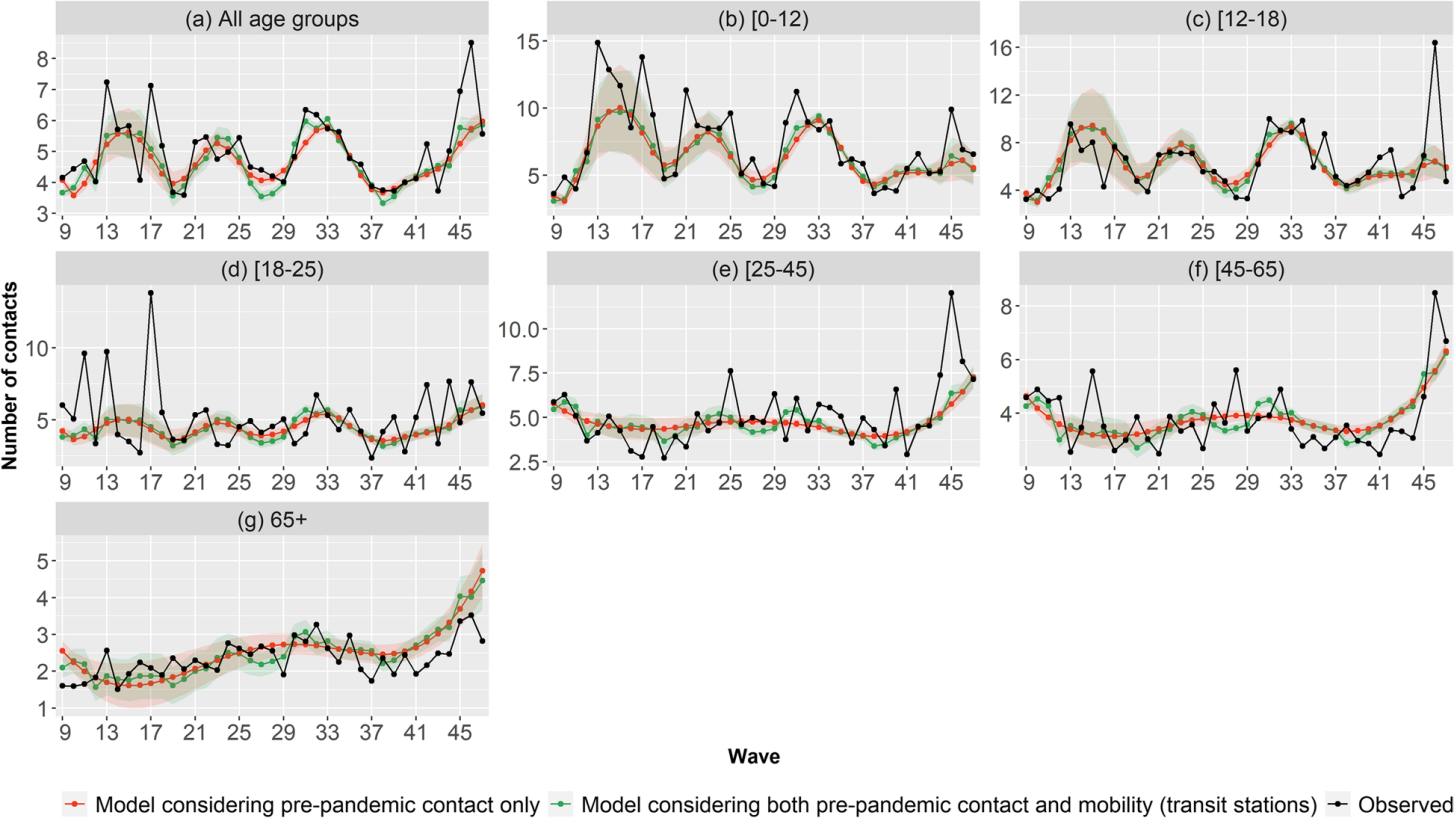
Total number of contacts per wave observed (black) and estimated (with IC-95) by the time varying model considering only pre-pandemic contacts (red) and both pre-pandemic contacts and Google mobility data at transit stations (green)

Figure 5 shows the observed (black) and estimated average number of contacts by wave by time varying effect model considering only pre-pandemic contacts (red) and considering mobility in addition (green), for each of the 39 waves considered in this study. The overall trend of the evolution of the number of contacts was well captured by the time-varying effect model considering only pre-pandemic contacts. Considering in addition mobility led to a similar fit. When looking at the estimation by age groups (Fig. 5 b-g), it can be seen that for all the age groups the fit is fairly good for both models. However, some sudden changes in the number of contacts are not well captured by both time-varying effect models. For example, the peaks in wave 45 for the age group [25-45) and in wave 46 for the age groups [12-18) and [45-65) are not predicted by both time varying effect models. Similarly, neither time varying effect model predicted the sudden peaks in the number of contacts observed in waves 11, 13, and 17 for age group [18-25). Confidence intervals are relatively narrow for all age groups throughout the study period, except between wave 12 and 20 for age groups [0-12) and [12-18).

## Discussion

In this study we modelled the social contact patterns observed between November 2020 and July 2022 [12, 26] based on social contact patterns before the pandemic [10, 11] through a time varying effect model. We then evaluated whether a publicly available proxy of mobility data from Google [27] could help to predict the time variation of the coefficients.

Firstly, we found that social contact interactions in pre-pandemic time seem to be a good starting point to predict contact patterns under NPIs. Although a study conducted in England between January 2020 and June 2021 showed that pre-pandemic and pandemic contacts pattern seem hardly similar to each other [32], it has been shown previously that some characteristics of pre-pandemic social patterns were similar compared to social contact patterns during the pandemic. For example, a study conducted in Canada [16] between March 2020 and February 2021 showed that typical mixing at home (i.e. assortative mixing and mixing between parents and children) seemed to hold during pandemic time. Therefore, considering social contact patterns at different locations is needed as it allows to take into account the locations in which the contact patterns are the most similar as compared as the pre-pandemic time. From our results, it seems that pre-pandemic contacts at home and school had the strongest association with the total number of contacts during the pandemic compared to the association observed for pre-pandemic contacts at work and other places. Interestingly, home corresponds to the location in which we observed a fairly similar social contact patterns as compared to the pre-pandemic time [16]. This result is expected as NPIs can not reduce contacts within a household. Pre-pandemic contacts at school seem also to be a good predictor of contacts for children under NPIs. As during the studied period, schools were mainly open [33], this observation was expected.

In a context in which social contact patterns are not yet available in real time, the use of a linear combination of pre-pandemic social contact patterns could prove valuable. However, the coefficients of the linear combination varied over time (Fig. 4). This was expected due to the varying intensity of the NPIs (Fig. 2) as well as the time variation in the number of contacts at work, school and other places (Fig. 3). The challenge of such an approach is then to translate the NPIs at a given time into appropriate coefficients.

To this extent, assuming that the time variations of the coefficients can somehow be related to aggregated mobility data seems not acceptable during our study period to estimate the number of contacts at a given time. Indeed, the time varying effect model considering both pre-pandemic contacts and mobility showed more variation in the coefficient functions than the one considering only pre-pandemic contacts (Fig. 4). This could be the sign that the correlation between mobility and the number of contacts vary over time. Therefore simply deriving changes in social contacts from mobility data can be overly simplistic [22]. When stringency of the NPIs evolves due to a change in the epidemiological context, people have to change their mobility (i.e. go back to work) while still attempting to minimise close contacts and maximise distance [22]. Zhang et al. [34] found that in China although mobility data can be a good proxy for social contacts during lockdown, this assumption does not hold in the post-lockdown periods. However they acknowledge that generalisation to other countries remains unclear. Another study based on data collected in 52 countries, found that although mobility correlates well with transmission intensity, this relationship is time dependent [35]. The temporal evolution of the dependency between mobility and total number of contacts could be related to the evolution of the risk perception towards COVID-19. Wu et al. [36] showed that perceived risk positively influences mobility. Moreover, [18] also showed that perceived risk positively influences the total number of contacts. However, even if both mobility and number of contacts are influenced by risk perception, the structure of the correlation may be different. Using changes in mobility patterns to estimate changes in the total number of contacts during a period in which perceived risk is evolving could therefore be unreliable. However, other approaches can avoid the need to estimate the coefficients of the linear combination. For instance use of GPS data from mobile phones to infer complex social networks could be another option to model the dynamics of transmission [23].

These results must be interpreted in the context of the following limitations. First, mobility data from Google only includes people using a smartphone and have enabled the location history setting [27]. This subset of people may not be representative of the whole population, and children and older people might be under-represented [37]. In addition, only a unique aggregated dataset is available so no personally identifiable information is available [27]. A study conducted in Japan based on non-publicly available data from Yahoo found that temporal changes in mobility were age dependent [38]. This finding may lead to bias in the estimation of our model considering mobility as we have used an average mobility value for all age groups. Similarly it is likely that variables other than age (e.g. socio-economic status, type of work, ...) influence both the number of contacts and mobility. However, in this study social contacts data were aggregated across age groups regardless of other variables. Our results apply at population level and may not be generalisable to specific sub-populations.

Another limitation of our work is that our model does not ensure positive predicted values. For a combination of values for which few contacts were observed, it can occur that the model predicted a small negative value for the number of contacts. However, none of the negative predictions were significantly different from zero. This is due to the hypothesis that the number of contacts is normally distributed. Hypothesizing a log-normal distribution would have ensured non-negative predictions. However, applying a log-normal distribution would have resulted in a less interpretable model. The interpretability of the model is facilitated greatly by the choice of the normal distribution for the outcome, and by a linear combination of pre-pandemic contacts, as has been done in previous models [5–8]. This is in line with our main research aim.

Moreover, the SAS package used does not provide a goodness of fit test for models using p-splines. Therefore, only a visual assessment of the goodness of fit was made. Although the fit looks good, this could be a limitation of our study.

Finally, for this study we only considered the change in the number of visitors to transit stations. Since change at this location may indicate mobility change at several locations, we considered it was the most appropriate to predict the overall change in contact patterns. However, mobility change at transit stations does not take into account the possibility that people may have switched from public to private transport (car, motorbike, bicycle, ....) while maintaining their overall contacts.

Another option would have been to consider mobility at work instead of mobility at transit stations. We could assume that homeworking decreases as mobility at work increases, while mobility at transit stations may not capture this. Mobility at transit stations may remain low because people avoid public transport to go to work. However, a disadvantage of using mobility at work is that mobility at work is very sensitive to holiday periods. During these periods, people are likely to have made contacts in places other than work, and this may be better captured by considering mobility at transit stations.

In order to determine what would have been the impact in term of coefficients functions and fit of using other mobility proxy, two sensitivity analyses were carried out. In the first analysis, we considered a model similar to the one in Eq. 4 while considering mobility at work instead of mobility at transit stations; in the second, we used average mobility across all settings, similar to what was done in [35]. For both sensitivity analyses, we found very similar coefficient functions (see Additional file 1: Figs. S1 and S2) and model fits (see Additional file 1: Figs. S3 and S4), compared to the analyses presented in the Result section. Nevertheless, it remains unknown whether our results can be generalised to mobility trends measured by data streams other than Google.

## Supplementary Information

**Additional file 1.**

## Data Availability

The social contacts data before the pandemic analysed for this study are available in [29]. The social contacts data during the pandemic (until survey wave 43) are available in the zenodo repository, [39]. The social contacts data during the pandemic after survey wave 43 are available in the zenodo repository, [40].

## Abbreviation

NPI: Non-Pharmaceutical Interventions
