# Supplementary material for: How contact patterns during the COVID-19 pandemic are related to pre-pandemic contact patterns and mobility trends
Source: BMC Infect Dis. 2023 Jun 16;23:410. doi: 10.1186/s12879-023-08369-8 (PMC10276431; doi:10.1186/s12879-023-08369-8)
Supplement: Supplementary file 1 — Additional file 1. [file 12879_2023_8369_MOESM1_ESM.pdf]

Supplementary material

Coefficient functions of the time-varying coefficient model considering mobility at work (Figure S1) and considering the average mobility across all settings (Figure S2) are very similar as the one considering mobility at transit stations (Figure 4). Similarly the estimation of the total number of contacts per wave of the model considering mobility at work (Figure S3) and considering the average mobility across all settings (Figure S4) are very similar as the prediction of the time varying model considering mobility at transit stations (Figure 5).

The 39 different waves of CoMix data collection considered in this work corresponding to waves 9 to 47 are summarised with 39 contact matrices and visualised in Figure S5.

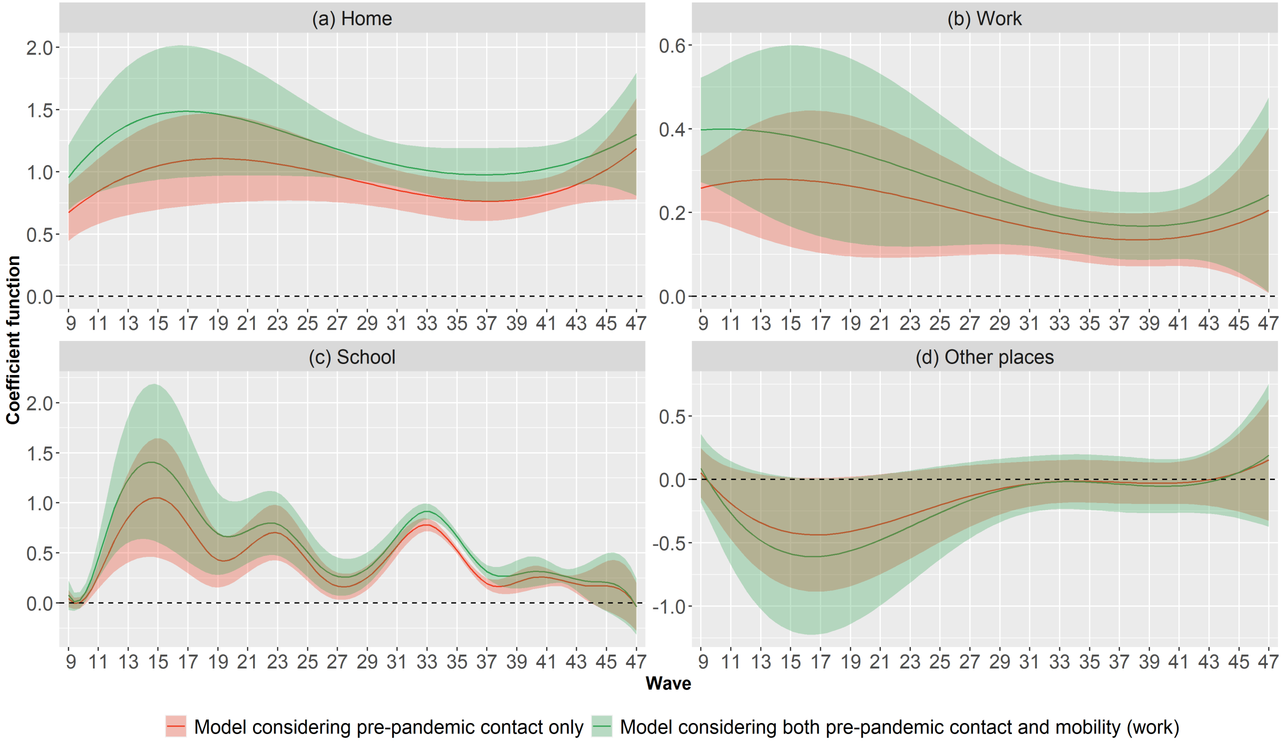

Figure S1: Coefficient functions of the time-varying coefficient models considering only pre-pandemic contacts (red) and considering both pre-pandemic contacts and Google mobility data at work (green).

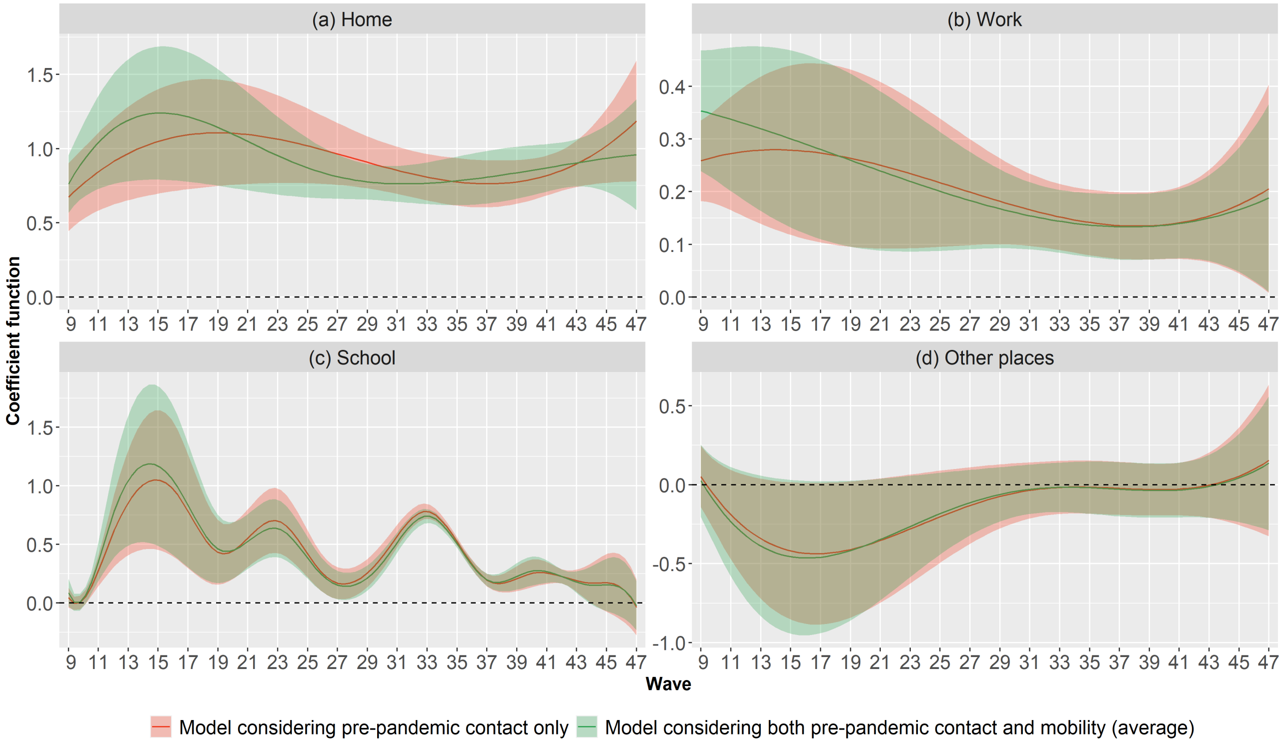

Figure S2: Coefficient functions of the time-varying coefficient models considering only pre-pandemic contacts (red) and considering both pre-pandemic contacts and Google mobility data (average across all settings) (green).

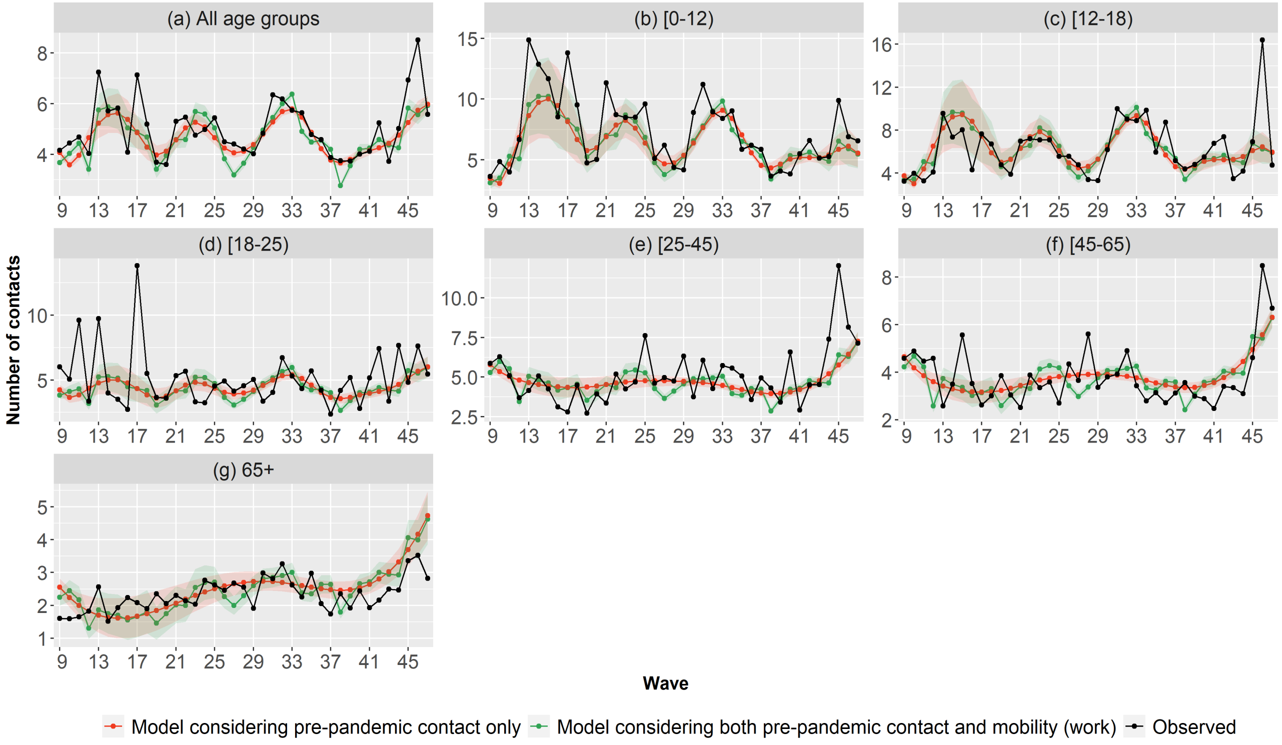

Figure S3: Total number of contacts per wave observed (black) and estimated (with IC-95) by the time varying model considering only pre-pandemic contacts (red) and both pre-pandemic contacts and mobility data at work (green).

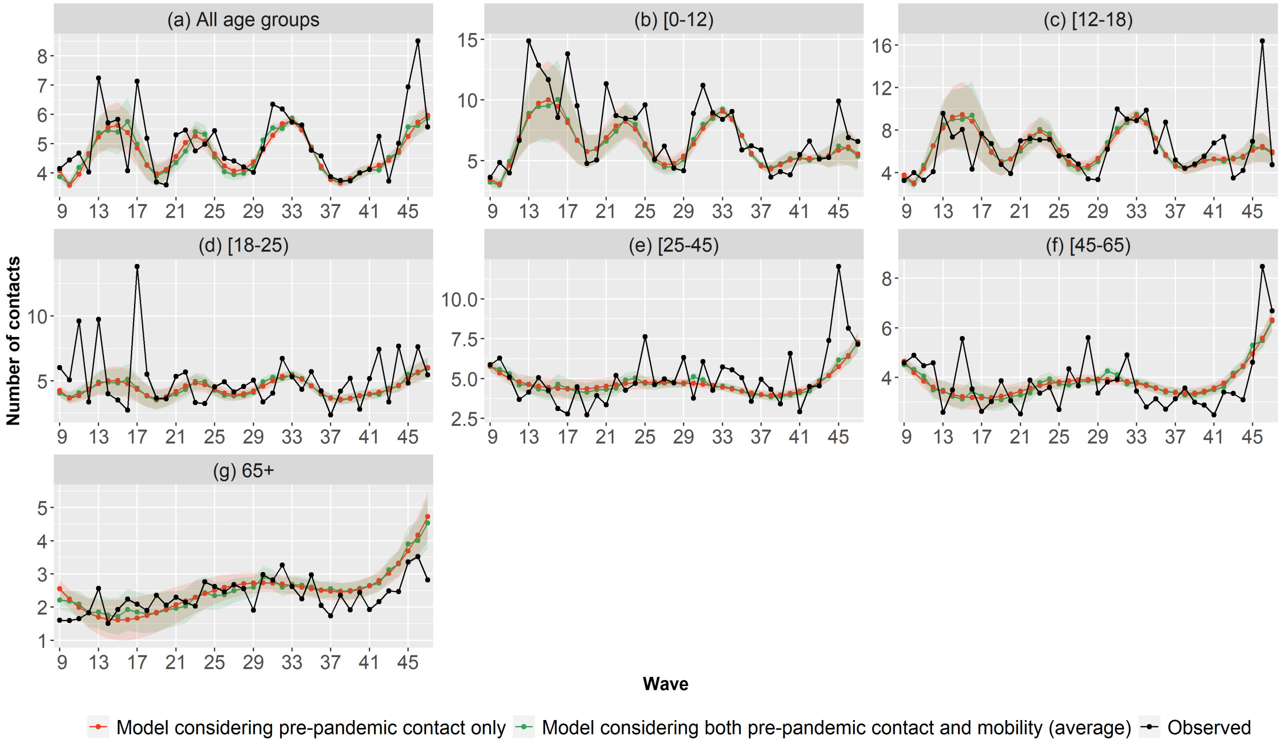

Figure S4: Total number of contacts per wave observed (black) and estimated (with IC-95) by the time varying model considering only pre-pandemic contacts (red) and both pre-pandemic contacts and mobility data (average across all settings) (green).

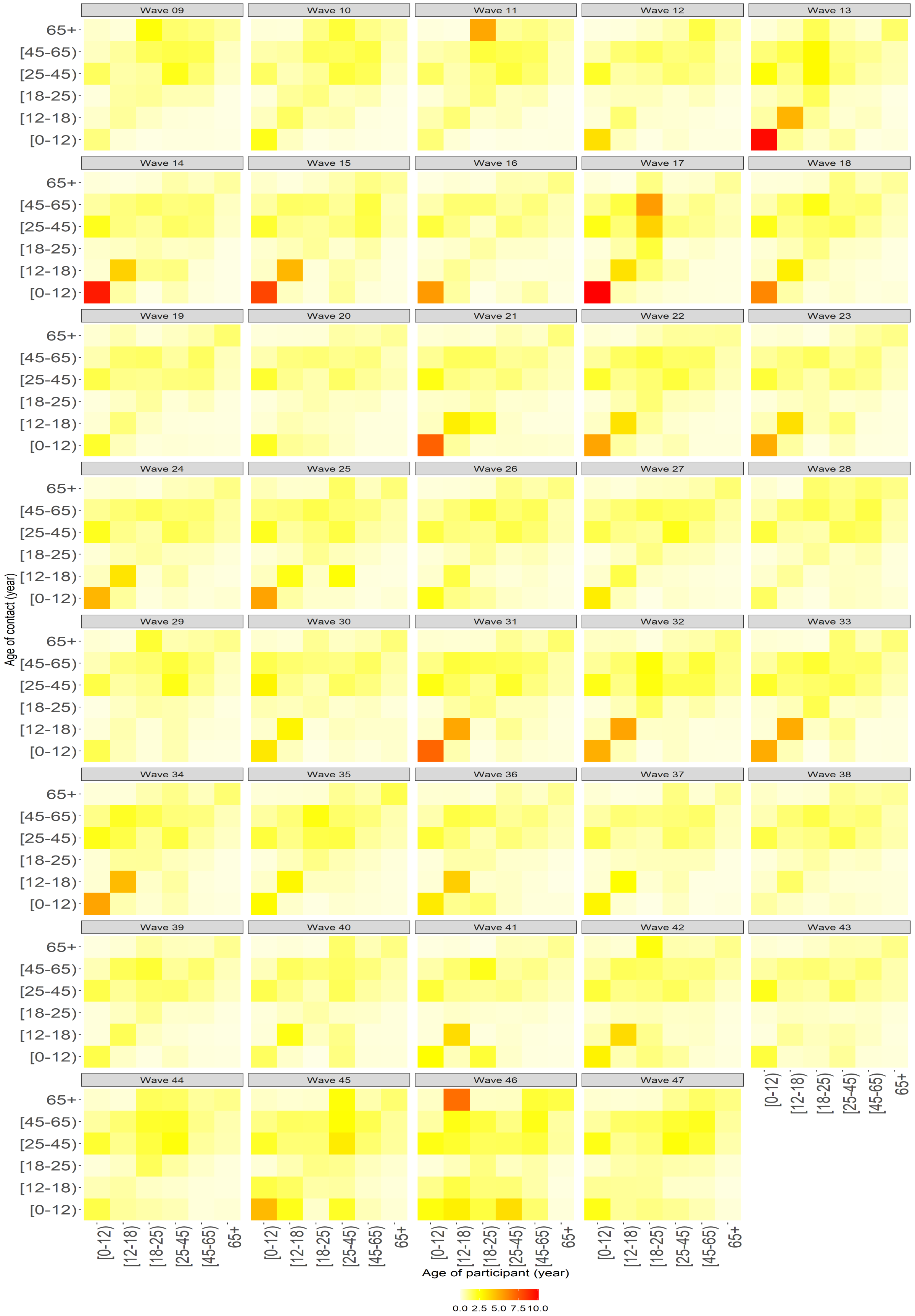

Figure S5: Contact matrices (average number of contacts per day) during pandemic times between November 2020 and July, 2022.
